# Molecular epidemiology, symptomatic analysis of anaplasmosis and associated study of milk reduction in cattle from district Nowshera, KP, Pakistan

**DOI:** 10.1186/s13620-025-00304-4

**Published:** 2025-12-01

**Authors:** Huma Fatima, Shawana Qayyum, Shazia Shams, Maryam Khan, Nargis Shaheen, Atiya Iqbal, Adil Khan, Ioannis A. Giantsis, Ayman A. Swelum

**Affiliations:** 1 Department of Zoology, Women University Mardan, Mardan, KP Pakistan; 2 Department of Zoology, Rawalpindi Women University, Rawalpindi, Punjab Pakistan; 3 Department of Zoology, Quaid i Azam University, Islamabad, Pakistan; 4 Department of Botany and Zoology, Bacha Khan University Charsadda, Khyber Pukhton Khwa Peshawar, Pakistan; 5 Department of Animal Science, Faculty of Agriculture, Forestry and Natural Environment, Aristotle University of Thessaloniki, Thessaloniki, Greece; 6 Department of Animal Production, College of Food and Agriculture Sciences, King Saud University, Riyadh, 11451 Saudi Arabia

**Keywords:** Prevalence, Anaplasmosis, Milk reduction, Cattle, Nowshera

## Abstract

*Anaplasma* is an obligate intracellular bacterium that cause anaplasmosis. The two species of *Anaplasma* namely *Anaplasma marginale* and *Anaplasma centrale* affects cattle, buffaloes, sheep, goats and wild ruminants. This rickettsial microorganism, which is transmitted by ticks, is causing massive economic losses due to weight loss, decreased milk supply, severe anemia and high mortality. The research study was conducted between May 2023 to April 2024 to determine the prevalence, symptomatic analysis of anaplasmosis and associated study of milk reduction in cattle from district Nowshera, KP, Pakistan.

Total 635 blood specimens were obtained from different cattle herds coming from 17 farms. Such blood specimens underwent microscopic examination by using Giemsa-stained blood smears and troughs molecular analysis by PCR targeting the msp1b gene (265 bp fragment) in order to detect *A. marginale*. All relevant statistical analyses, including Chi-square tests, were carried out with SPSS to test for significance (*p* < 0.05). Combining microscopy and PCR, the overall prevalence of *A. marginale* was determined to be 13.3% (*n* = 85) and 12.7% (*n* = 81) respectively. Age-wise prevalence revealed that younger cattle (< 5 years) had significantly higher infection rates 62.3% (*n* = 53) as compared to older cattle (> 5 years) 37.6% (*n* = 32), with a *p*-value of 0.037. Of the PCR-positive cases, 64.1% (*n* = 52) were from younger cattle and 35.8% (*n* = 29) were from older cattle. A potential cause for the relatively low PCR positivity in comparison to microscopic identification is that certain false-positive microscopic identifications based on Heinz bodies or other blood artifacts as *Anaplasma* species may be involved. Naturally, PCR would only target the genetic material of the actual disease. Microscopy showed that prevalence peaked in the month of July at 24.7%, while PCR recorded peak prevalence in June at 33.3%. There were no confirmed cases in January and February, confirming that the differences in seasonal prevalence were statistically significant (*p* < 0.05). In terms of production loss, one-year-old Holstein cows experienced a 47% reduction in milk yield post-infection (from 17 L to 9 L), while cows 2 to 7 years-old exhibited reductions ranging from 50 to 75%, all statistically significant. It was concluded that anaplasmosis was widely distributed in district Nowshera and it is high economic losses on dairy farm. Further research is needed to develop control measures and to improve understanding of the disease transmission.

## Introduction

*Anaplasma* is an obligate intracellular bacteria being a part of Anaplasmataceae, order: Rickettsiales, species: *A. marginale* and *A. centrale*. Anaplasmosis is a term for a disease caused on by the *Anaplasma* species [1]. Gall sickness is another name for Anaplasmosis. Common hosts of anaplasmosis includes cattle, buffaloes, sheep, goats and wild ruminants [1]. This rickettsial microorganism, which is transmitted by ticks, Tabanus and Stomoxys, is causing massive economic losses due to weight loss, decreased milk supply, severe anaemia and high mortality. *Anaplasma* infections are more common in cattle than in buffaloes [1]. The developmental cycle of *A. marginale* in ticks is sophisticated and synchronised with their consuming food cycle [2, 3]. The intestinal cells of ticks are infected by *A. marginale* after digesting infected red blood cells ingested by ticks during a blood meal. After the pathogen’s growth in tick gut cells, the infection spread to numerous tick tissues, including the glands of saliva, where cattle receive their food [4, 5]. At every inoculation site, *A. marginale* grows inside communities or vacuoles that are membrane bounded. The first form of *A. marginale* to be seen within the colony is the reticulated (vegetative) form. It divides by binary fission to generate enormous colonies that may contain 100 of organisms. After that, the reticulated form transforms into the dense form, which is the contagious type and has a brief half life outside of host cells [5].

Anaplasmosis in cattle might manifest as any of the following symptoms: Sudden death, Acute anemia, fever, weakness, breathing difficulty, depression, reduced appetite, bloating, blood in the urine or hematuria, jaundice (yellowing of skin), abortion [6]. A study by Sajid et al., in a district Khanewal falling in Punjab reported the prevalence of anaplasmosis as 4.17% [7]. Shaukat et al. report 10.84% prevalence of bovine anaplasmosis in Sahiwal and crossbreed cattle of district Faisalabad, Punjab, Pakistan [8]. Atif et al. reported an incidence of 9.71% for the anaplasmosis infection in Sargodha district, Pakistan [9]. Asif et al. recorded an 11.1% prevalence rate of anaplasmosis infection in cattle from district Multan, Southern Punjab, Pakistan [10]. According to Atif et al., a maximum prevalence of *A. marginale* in the Sargodha district (37.1%) was followed by the districts Khushab (31.4%) and Rawalpindi (24.5%) of northern Punjab, Pakistan [11]. Jaimes-Dueñez et al. report a 54.8% prevalence of *A. marginale* infection in cattle from Colombia, America [12]. A study conducted by Isik et al. reported an 11.2% prevalence of *A. marginale* in cattle in the province of Konya, Turkey [13].

In some regions, for more than 30 years, imido-carb dipropionate has been utilized to treat the illness [14]. Laboratory investigations, such as light microscopic examination of stained blood smears or serological/molecular diagnostics methods, are necessary to confirm the diagnosis [15].

Using the light microscopy, the thin blood smears stained with Geimsa, wright Geimsa, or diff stain. The use of quick stain may make it easier to see *A. marginale* organism in the erythrocytes. But in circumstances where the sickness is more advanced and the animals have severe anaemia, this method might not work [16]. Due to their great sensitivity and specificity, serological and molecular diagnostics are the techniques that can detect *A. marginale* with ore specifically [17]. It has been advised to use Polymerase Chain Reaction (PCR), which is based on the amplification of DNA fragments, to identify infections in animals that are going to be sold and/or transported abroad [18]. After recovering from an acute infection, blood smears obtained from animals that are continuously infected usually do not show *A.marginale* inclusions. Several ruminants, including cattle, sheep, and deer, have had *A.marginale* infections diagnosed using a competitive ELISA [19–22]. Knowles et al., created the ELISA that is presently used to diagnosed bovine anaplasmosis [20]. It is based on the use of a monoclonal antibody (Mab) ANAF16C1, which recognises msp5 in *A. marginale*, *A. centrale* and *A. Ovis* [23]. Since the msp5 sequence is largely conserved, strains of *A.marginale* as well as *A. phagocytophilum*, *A. centrale*, and *A. marginale* are similar to one another. The identification of comman areas determined to be required for ANAF16C1 reactivity has confirmed the cross-reactivity of the msp5 test with multiple species of *Anaplasma* [24]. Because of this, the msp5 ELISA as unable to distinguish between distinct species of *Anaplasma* in geographical areas where co-infection with a *A. phagocytophilum* and *A. marginale* or *A. centrale* occurs [21, 25, 26]. Since its development by Morzaria et al., [27] Svanova Biotech AB (Uppsala, Sweden) has been selling an ELISA based on recombinant MSP5 for the indirect detection of *A. marginale* antibodies. However, the assays cross-reactivity with additional *Anaplasma* species has not been assessed. To diagnosed *Anaplasma* infections, DNA-based diagnostic techniques have been created. However, as of currently, the most feasible way of testing a large of number of cattle for signs of illness is still using a serologic test based on msps [27].

## Objectives of the study

To find out the overall prevalence of *A. marginale* in cattle from district Nowshera, KP, Pakistan.

To compare the age wise prevalence of *A. marginale* in cattle from district Nowshera, KP, Pakistan.

To find out the month wise prevalence of *A. marginale* in cattle from district Nowshera, KP, Pakistan.

To determine the milk reduction in cattle due to *A. marginale* in district Nowshera, KP, Pakistan.

## Materials and methods

### Study area

**Fig. 1:**
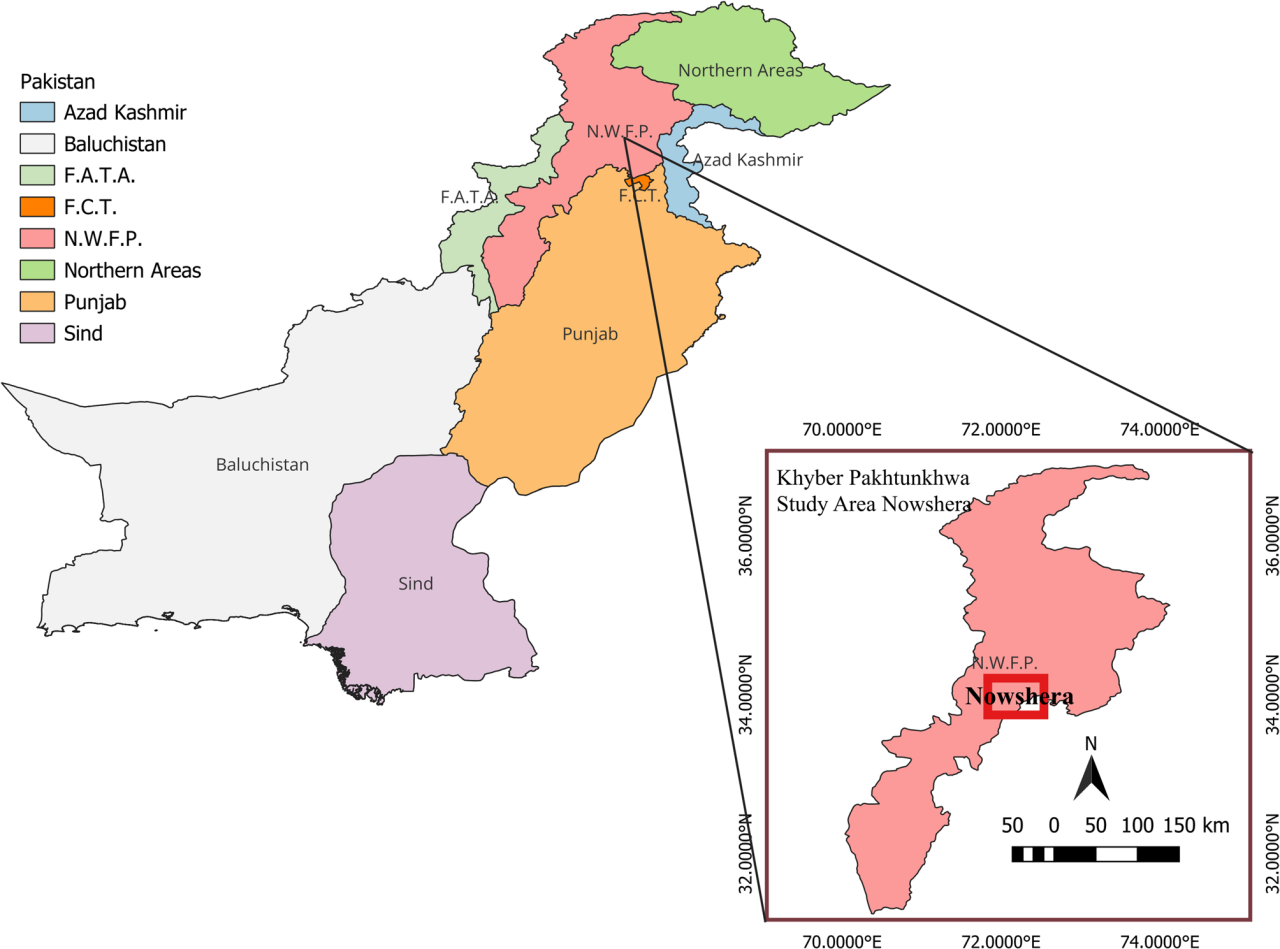
Map showing study area

Between May 2023 to April 2024, a cross sectional study was carried out in district Nowshera in order to determine the prevalence, symptomatic analysis of anaplasmosis and associated study of milk reduction in cattle (Fig. 1).

Nowshera district is located in Khyber Pakhtunkhwa province, Pakistan, between 33° 42 to 34° 09 North latitude and 71° 97 to 72° 15 East longitude, at an altitude of 285 m (935.04 feet) above sea level [28].

According to Weather and Climate [29], Nowshera experiences a humid subtropical climate (Cfa) without a dry season according to the Koppen classification. The district average high temperature throughout the year is 23.23°С (73.81 °F), and the average low temperature is 11.47°С (52.65 °F). Annually, Nowshera receives approximately 131.34 millimeters (5.17 inches) of rainfall, with rain occurring on about 145.14 days, or 39.76% of the year. This climate is favourable to the survival and transmission of *A. marginale* [29].

The livestock grazing areas in Nowshera are mainly located on farms including Amin dairy farm (Azakhel payan), Zaman and Ahmad farm (Bara banda), Hamza Khan dairy farm (Surya khel), Khattak dairy farm (shaidu), Durrani farm (Watter), Nowshera cattle farm (Hakimabad), Pakistan holstein (Dairy farm Risalpur), and others. Cattle on these farms graze in open or semi-closed areas, which could transmit *A. marginale*.

The vegetation primarily consists of grasses and shrubs. This provides forage for livestock including cattle that might be susceptible to *A. marginale*, which provide favourable habitats for ticks, the primary vector of *A. marginale*.

### Sample size

The total Six hundred and thirty five (635) samples of cattle were collected across all farms, the average number of animals sampled per farm varied, but 37 samples were taken from each farm. The study included 17 cattle farms located in Nowshera district. Only a few farms used modern farming methods, the majority were traditional in nature. Some of cattle at these farms got feed supplementation, but most were raised using conventional methods. Both healthy and disease cattle, were used in the investigation to determine the presence of *A. marginale*. In order to ensure a targeted sample for the prevalence investigation, cattle were randomly selected from each farm, regardless of their age, breed and health status. The demographic information, including age (reported in years), sex, breed, and lactation status, were provided by the farmers.

Clinical signs associated with *A. marginale* infection such as fever, lethargy, loss of body weight, pale mucous membrane, breathing difficulty, depression, reduced appetite, bloating, blood in the urine or hematuria, Jaundice (yellowing of skin), reduced milk production, and anaemia were also collected along with demographic data on the affected animals and noted based on physical examination as well as farmers observation. This symptomatic data was necessary to support the diagnosis through microscopy and to evaluate the relationship of clinical signs with infection status.

### Demographic investigation of *A. marginale* infection in cattle

To assess the level of infection, the microscopy and molecular prevalence were evaluated. The microscopy-based prevalence was calculated according to Rony et al. with the following formula: [30]

$$\mathrm{Microscopy}-\text{Based Prevalence}\left(\%\right)=\left(\frac{\text{Number of microscopically positive samples}}{\text{Total number of samples examined}}\right)\times\:100$$

According to Khattak et al., the prevalence on the basis of molecular diagnosis was calculated [31].

$$\mathrm{Prevalence}=\frac{\text{PCR positive cases}}{\text{Total suspected cases}}\:\times\:100$$

### Collection and transportation of blood samples

To detect *A. marginale*, blood samples (3-5 ml) were obtained from the jugular vein. Prior to drawn blood from the jugular vein, 70% alcohol was used to disinfect the puncture site. Blood specimens were collected from study animal’s jugular vein using sterile disposable needle (10 ml/cc) and placed in EDTA containing vacutainer. After labelling, the collected samples of blood were brought, to the Rural Health Centre Pirpiai Lab, Nowshera in an ice box for the preparation of blood smears. The samples were then stored at 4 °C until further molecular analysis.

### Preparation and microscopic examination of blood smears

To detect *A. marginale*, blood was collected from the jugular vein of each cattle and used to prepare thin blood smears. The procedure involved applying a drop of blood on to a cleaned slide at a 45°angle, and then proceed ahead slowly, as explained by Kessell [32]. The smears were fixed in methanol, labelled with a sample code, and allowed to air dry. For staining the slides were immersed in 10% Giemsa stain for 20 to 25 min, then washed with tap water and air-dried. The stained smears were examined using a Zeiss Primo Star light microscope (Carl Zeiss Microscopy GmbH, Germany) at 100X magnification with immersion oil. *Anaplasma* species were identified as intraerythrocytic inclusions measuring 0.3–1 μm in diameter, with a blue-violet stained. *A. marginale* was specifically observed along the edges of erythrocytes. Infected cattle were considered positive if one or more intraerythrocytic inclusions were present, as stated by Ziam et al. [33].

### DNA extraction from the cattle blood

Using an inorganic method, DNA extraction from cattle blood was performed. Several reagents including 1 M Tris HCl (pH 8), 0.5 M EDTA (pH 8), TE buffer, TNE buffer, 5 M NaCl, 10% SDS, 70% ethanol, and 20 mg/ml Proteinase K were prepared. Blood samples were kept frozen at −20 °C until thawed at 37 °C, and then 500 µl was used for the processing with TE buffer with repeated centrifugation and washing for hemoglobin removal. The pellet was treated with TNE, SDS, and proteinase K following incubation overnight at 37 °C or for 2 h at 50 °C. After protein precipitation with 5 M NaCl, inactivation of the enzymes on ice, DNA was precipitated with chilled isopropanol, centrifuged, washed twice with 70% ethanol, dried, and dissolved in 50 µl TE buffer. Tubes were kept in hot water for 1 h at 70 degrees Celsius to inactivate enzymes before storage at −20 °C. A 0.8% agarose gel electrophoresis using 1X TBE buffer, ethidium bromide staining, and visualization under UV light using a Biostep illuminator confirmed the presence of DNA.

### Polymerase chain reaction (PCR)

**Fig. 2:**
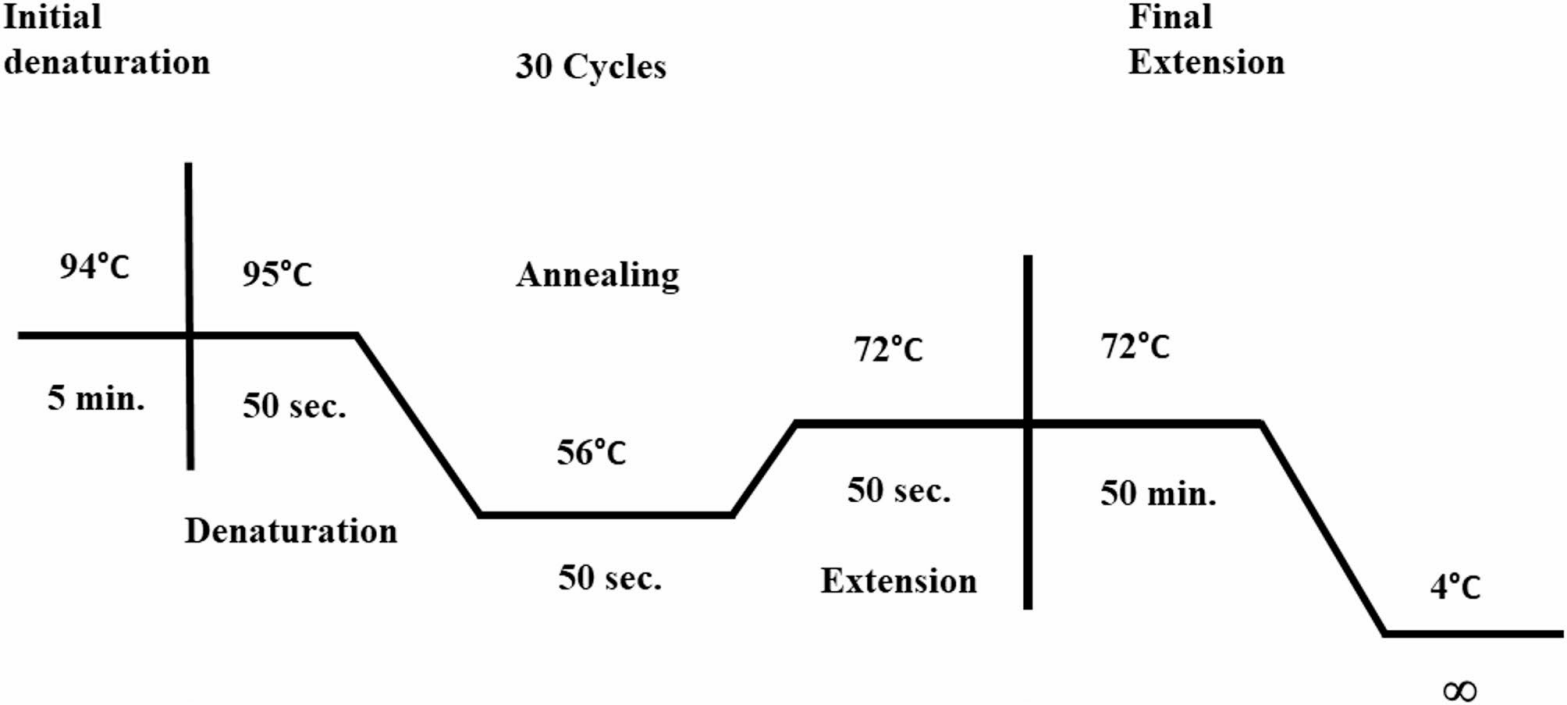
Thermal cycler conditions for the PCR based amplification of msp1b gene of *A. marginale* in the cattle blood samples collected during present investigation (Bilgic et al., [34])

According to Bilgic et al. [34], a 265 bp fragment primer set for msp1b, the major surface protein 1b gene of *A. marginale*, has been applied. The forward primer sequence is 5’-GCTCTAGCAGGTTATGCGTC-3’ whereas the reverse one is 5’-CTGCTTGGGAGAATGCACCT-3’. PCR was performed with 5 µL of template DNA, 5 µL of 10X PCR buffer, 4 mM of MgCl₂, 2 mM of each dNTP, 2 µM of each primer, and 1.5 units of Taq DNA polymerase (AbClonal, USA), in a final volume of 50 µL made up with double-distilled water. The PCR amplification was done in the Gene Amp PCR System 2700 thermal cycler (Applied Biosystems Inc., UK) after initial denaturation at 94 °C for 5 min, followed by 30 cycles of denaturation at 95 °C for 50 s, annealing at 56 °C for 50 s, and extension at 72 °C for 1 min, and a final extension at 72 °C for 5 min (Fig. 2) [34]. The PCR products were run on an agarose gel containing 1.8% agarose obtained by dissolving 2.48 g of agarose in 135 mL of 1x TBE buffer, followed by estimation of the DNA size using a 100 bp DNA ladder (New England Bio Labs, USA).

### Quantifying losses in milk production

Different ages of cows showed variable degrees of milk reduction, after infection with *Anapalsma marginale*. Seven-year old cows showed the largest decrease in milk yield (75%), while One-year old cows showed the least decrease (47.0%). The milk reduction period varied by age, with 1-year old cows experiencing it for 1 week and 7-year old cows experiencing it for 14 weeks. The Amount of milk produced decreased by 56.2% from 192 L before infection to 84 L after infection. This decrease, which has a *p*-value of 0.0002, statistically significant and indicates that cow milk output is impacted by *A. marginale*.

### Statistical analysis

A statistical analysis was conducted on 635 cattle to determine the prevalence of *A. marginale* infection. Descriptive statistics were used to summarize the data. Microsoft Excel (version 2019) and IBM SPSS (version 26) were used for data entry and analysis. In order to compare infection status with symptoms of concern, the Chi-square test was utilized. The statistically significant difference in infection rates was shown by a *p*-value of less than 0.05, indicating that the frequency of anaplasmosis is not random but rather represents a noteworthy trend in the community being studied.

## Results

### Prevalence (%) of *A. marginale*

**Fig. 3:**
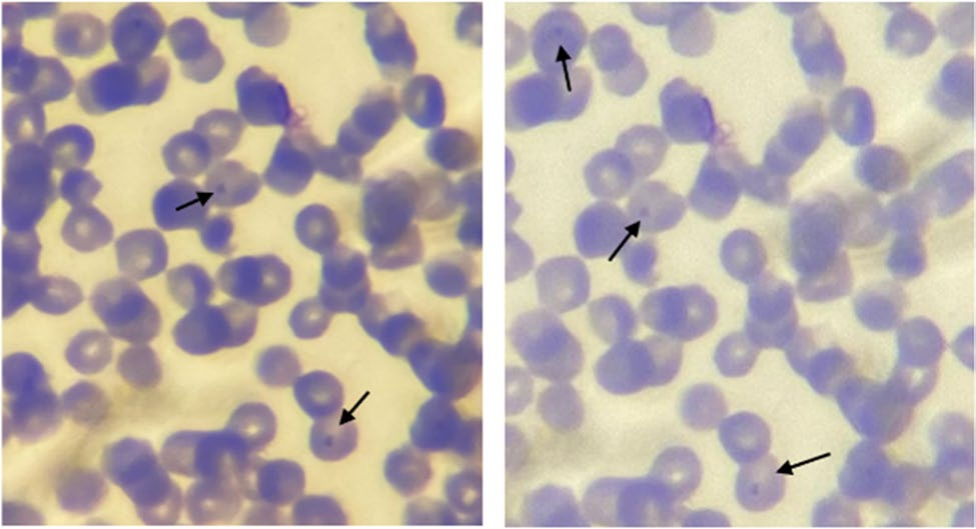
Intraerythrocytic bacteria appear spherical and are located at the edge of RBCs (*A. marginale*)

**Fig. 4:**
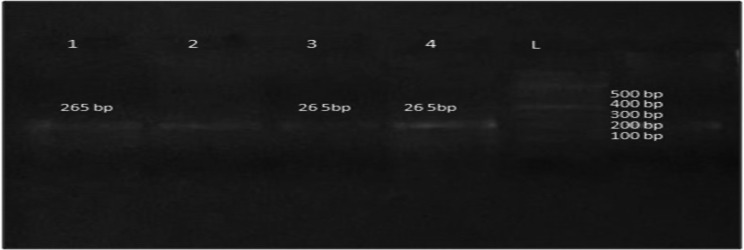
PCR based amplification of msp1b gene of *A. marginale* in cattle blood samples that were collected during present study. Lane: L represent 100 bp DNA marker, lanes 1, 2, 3 and 4 represent *A. marginale* positive blood *samples*

*A. marginale* were observed under the microscope as stained blue violet intraerythrocytic inclusion found along the margin of red blood cells (Fig. 3). The identification was based on the clinical condition of the cattle and confirmed through blood smear examination and Polymerase chain reaction. Polymerase Chain Reaction amplified a 265 bp fragment specific for msp1b gene of *A. marginale* (Fig. 4). PCR amplification was carried out on all microscopy-positive samples to confirmed the diagnosis.

A total 635 cattle were examined from May 2023, to April 2024. Out of these, 13.3% (*n* = 85) were positive for *A. marginale* infection by microscopy and 12.7% (*n* = 81) by molecular basis (Table 1).

**Table 1:** Overall and age wise prevalence of *A. marginale* in cattle of district Nowshera

Factor (Prevalence)	Total Examined Cases (*n*)	Total Negative Cases (*n*)	Microscopy Positive Cases (%)	PCR Confirmed Cases (%)	*p*-Value
Anaplasmosis in Cattle	635	550	85 (13.3)	81 (12.7)	*p* = 0.037
Younger Cattle (< 5 years)	329	276	53 (62.3)	52 (64.1)	
Older Cattle (> 5 years)	306	274	32 (37.6)	29 (35.8)	
Total	635	550	85 (100)	81 (100)	

The Age wise prevalence rate of tick-borne disease was 62.3% (*n* = 53) in younger cattle while 37.6% (*n* = 32) in older cattle by Microscopy (Table 1). While the PCR amplification confirmed 64.1% (*n* = 52) positive cases in younger cattle and 35.8% (*n* = 29) positive cases in older cattle. The prevalence of anaplasmosis and the animal age has a significant difference (*p* < 0.05) and the *p*-value was 0.037, according to the results of the chi-square analysis. However, microscopy gave a higher number of positives than PCR, indicating a chance of false positivity with microscopy. The discrepancies may arise from the degradation of DNA from the blood samples, the inhibition of PCR by components in the blood, or misidentification of similar structures such as Heinz bodies. Hence, PCR remained very important in confirming its true identity, thus lowering chances for errors in diagnosis and effectively differentiating *Anaplasma* from other such look-alike structures.

### Month wise prevalence (%) of *A. marginale*

**Fig. 5:**
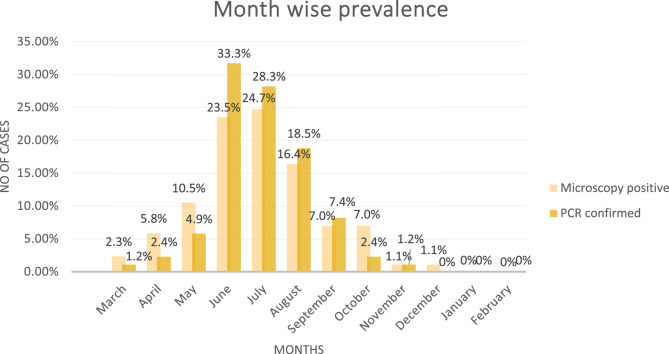
Month wise prevalence of *A. marginale* in cattle of district Nowshera

The current study indicates that July has the highest microscopy-positive prevalence (24.7%), with June having the next highest prevalence (23.5%) and in the rest of months prevalence recorded in August (16.4%), May (10.5%), September (7.0%), October (7.0%), April (5.8%), March (2.3%). However, November (1.1%) and December (1.1%) had the lowest prevalence was recorded and no cases were reported in January and February. Using PCR to confirm the positive samples yielded a slightly different pattern from that of microscopy; the highest PCR-confirmed prevalence was in June (33.3%) and July (28.3%), followed by August (18.5%), then May (4.9%) and September (7.4%), while April and October demonstrated (2.4%) each and for March and November only (1.2%) each, whereas December, January, and February showed no confirmed case at all. The *p*-value was 0.000. Significant difference at *p* < 0.05 (Fig. 5).

#### Statistical analysis result

The statistical analysis showed that there is a significant difference in the *A. marginale* infection rate from month to month. The Chi-Square test was employed in assessing the differences, producing a *p*-value of 0.000, which is statistically significant at *p* < 0.05. This indicates that the differences in month-wise prevalence levels observed are probably not due to random chance. Microscopy showed that, the peak prevalence was noted in July at 24.7%, followed by June at 23.5%, while PCR showed peak prevalence in June at 33.3% followed by July 28.3% and there was a sharp decline after that in the following months. The lowest Anaplasmosis prevalence was seen in November and December at 1.1% by microscopy, and no cases were reported in January and February.

### Milk reduction in infected cows

The infected cattle were currently exhibiting signs of decreased milk production. A simple percentage calculation method was used to quantify the loss of milk resulting from *A. marginale* infection. The milk production of the infected cows was compared to their milk production prior to infection, using the following formula:

$$\text{Milk reduction percentage}=\left(\frac{\text{Milk yield before infection-Milk yield after infection}}{\text{Milk yield before infection}}\right)\times\:100$$

Such method is widely used in veterinary research to quantify the effects of infectious diseases on dairy production, with modification of the relevant disease and type of cattle [35].

The amount of milk produced by the positive cow was lower than that of *A. marginale* negative cow. Prior to infection, the studied cows yielded 192 L of milk, after infection with anaplasmosis the milk production in cows was reduced to 84 L. The 1-year cows was produced 17 L of milk after infection with anaplasmosis it reduced to 9 L (47.0%). Similarly, the 2 years (20 L), 3 years (30 L), 4 years (40 L), 5 years (35 L), 6 years (30 L) and 7 years (20 L) cows milk production were at peak while infection with *A. marginale* it reduced to 10 (50%), 15 (50%), 20 (50%), 10 (71.4%), 15 (50%), and 5 (75%) respectively. The *p*-value was 0.0002. Significant at *p* < 0.05 (Table 2).

**Table 2:** Milk reduction in infected cows

Factor (Age of cows)	Number of cows (*n*)	Duration of milk reduction	Milk yield before infection (L)	Milk yield After infection (L)	Percentage (%)	*p*-value
1 year	15	1 week	17	9	47.0%	*p* = 0.0002
2 years	23	3 weeks	20	10	50%	
3 years	8	5 weeks	30	15	50%	
4 years	5	6 weeks	40	20	50%	
5 years	2	8 weeks	35	10	71.4%	
6 years	1	5 weeks	30	15	50%	
7 years	2	14 weeks	20	5	75%	
Total	56	42	192	84	56.2%	

## Discussion

Bovine anaplasmosis (BA), triggered by *A. marginale*, is a widespread tick-borne disease with significant financial implication for the cattle industry across various regions such as Asia, Africa, Australia, Southern Europe, and Central and South America [36], Its transmission occurs biologically through Rhipicephalus ticks and mechanically through flies, tainted needles, and farm equipment (such as Syringe, ear tags and other tools). Animal husbandry plays a crucial role in Pakistan’s economy, acting as the main origin of livelihood and support for eight million rural households.

In Pakistan, as a predominantly agricultural country, the animal husbandry field has made significant advancements, representing 58.9% of the agricultural industries. While the cattle business holds significant economic importance, it encounters notable obstacles stemming from both infectious and non-infectious diseases. Highly contagious tick-borne illnessess particularly strain farmers financially, with anaplasmosis being a widespread bacterial disease among domestic animals, leading to significant economic losses [37].

The total 635 samples collected from the Nowshera district, 13.3% (*n* = 85) blood samples of cattle that tested positive for *A. marginale* through microscopy and 12.7% (*n* = 81) by PCR. The samples were chosen on the observation of clinical signs and symptoms of *Anaplasma* infection, which called for further testing to confirm the presence of the pathogen.

This study shows that the prevalence of anaplasmosis in Nowshera district is 13.3% and 12.7% confirmed by Microscopy and PCR respectively. This rate is higher than the 6.1% prevalence reported in three Tehsils (Charsadda, Tangi and Shabqadar) of the Charsadda-Lakki district [38] but lower than the 19.6% prevalence observed in Marwat district [39].

According to Zeb et al. [40],. *A. marginale* was found to be 16.3% prevalent based on PCR data. A district-by-district research showed that the pathogen was most common in district Chitral (10.3%) and least common in district Dir Upper (13.6%) [40]. In Multan district, the overall, the *A. marginale* prevalencrate was estimated to be 11.1% (113/1020) of the examined cattle samples based on PCR amplification of the msp5 partial sequence [41].The epidemiological distribution of *Anaplasma marginale* in North Central Morocco, the overall prevalence of *A. marginale* was 21.9% by nPCR [42]. In China, the overall prevalence of *A. marginale* was 31.6%, when the blood samples from cattle that were subjected to PCR using the msp4 gene [43].

In present study the high rate of *A. marginale* infection was observed in cattle younger than 5 years (62.3%) as compared to the cattle older than 5 years (37.6%). While the PCR amplification confirmed positive cases, 64.1% (*n* = 52) were from younger cattle and 35.8% (*n* = 29) were from older cattle. A potential cause for the relatively low PCR positivity in comparison to microscopic identification is that certain false-positive microscopic identifications based on Heinz bodies or other blood artifacts as *Anaplasma* species may be involved. Naturally, PCR would only target the genetic material of the actual disease.

According to Radwan et al. [44], the erythrocytes of 26 of the 40 negative blood samples had *Anaplasma*-like features. Because *Anaplasma* species and structures such as Heinz bodies, Howell-Jolly bodies, or staining artifacts which are frequently observed in Giemsa-stained blood smears are difficult to distinguish, PCR was used to evaluate the DNA from the relevant blood samples. *A. marginale* was found using the PCR technique in 26 of 40 blood samples. High conservation was found among 26 PCR amplicon sequences from spontaneously infected cattle, according to msp5 sequence analysis [44].

The rise in occurrence can be linked to the comparatively less robust immune system found in young cattle. Conversely, Cattle that was older than five years old exhibit enhanced immunity, which inherently increases their resistance to a range of illnesses. This correlation underscores the significance of age-related immunity in shaping vulnerability to diseases and prevalence trends. A study conducted by Badshah et al., reported the young cattle (< 2 years) exhibits a greater prevalence of anaplasmosis (26.7%) compared to adults (> 5 years), which had a prevalence of (12.3%) in the Bannu, Lakki Marwat districts of KP, Pakistan [45]. Khan et al., recorded the younger cattle (< 5 year) showed highest prevalence of Anaplasmosis (24.8%) than older cattle (> 5 year) showed (13.1%) in Southern Khyber Pakhtunkhwa, Pakistan [39]. According to Vetrivel et al., observed that the incidence rate was lower in cattle older than five years (16.6%) and greater in cattle younger than three years (22.9%) in the districts (Kancheepuram, Tiruvannamalai and Vellore) of North Eastern zone of Tamil Nadu [46].

The months wise distributions showed that prevalence of anaplasmosis infection high in the month of July and incidence were reported (24%), the second highest prevalence rate of *A.marginale* infection was observed in June (23.5%) and the rest of the months prevalence recorded in August (16.4%), May (16%), September (7.0%), October (7.0%), April (5.8%), and March (2.3%). While the least incidence was recorded in the month of November (1.1%) and Dec (1.1%), and no cases was reported in January (0%), and February (0%). A slightly different pattern was obtained when PCR was used to confirm the positive samples than when microscopy was used; the highest PCR-confirmed prevalence was in June (33.3%) and July (28.3%), followed by August (18.5%), May (4.9%), and September (7.4%). In contrast, December, January, and February showed no confirmed cases at all, while April and October showed (2.4%) and March and November only (1.2%). This finding are also in line with the observations reported by Khan et al., reported the greater prevalence of *Anaplasma* infection in the month of July (13.3%), while the lowest prevalence in Jan, Feb, Mar, Nov and Dec (0%) in district Charsadda, Khyber Pakhtunkhwa, Pakistan [38].

According to Khan et al., the greatest monthly prevalence was noted in June (38%), with May following closely behind (34%), the March (10%) and January (8%). February had the lowest prevalence at 6% at Lakki Marwat district, KP, Pakistan [39]. A study conducted by Badshah et al., observed the highest occurrence in June (45.7%), while the lowest was recorded in February at 3.5% in Bannu and Lakki Marwat district [45].

In this research findings are corroborated by Taylor et al., who noted the prevalence of *A. marginale* across all seasons, with the highest occurrences during the warmer months (May, June, July, August, September, October) due to the conducive hot and humid environment for insect and tick development and reproduction [47].

In this study the anaplasmosis infection causes milk reduction in cattle, the amount of milk produced by the positive cow was lower than that of *A. marginale* negative cow. A simple percentage calculation method was used to quantify the loss of milk resulting from *A. marginale* infection. Prior to infection, the studied cows yielded 192 L of milk, after infection with anaplasmosis the milk production in cows was reduced to 84 L. The 1-year old cows was produced 17 L of milk after infection with anaplasmosis it reduced to 9 L (47.0%). Similarly, the 2 years (20 L), 3 years (30 L), 4 years (40 L), 5 years (35 L), 6 years (30 L) and 7 years (20 L) cows milk production were at peak while infection with *A. marginale* it reduced to 10 L (50%), 15 L (50%), 20 L (50%), 10 L (71.4%), 15 L (50%), and 5 L (75%) respectively. The *p*-value was 0.0002. Significant at *p* < 0.05.

The reason of milk reduction in cows, the anaplasmosis may result in anaemia, leading to harm to different body tissues and organs, such as the mammary gland. Moreover, the bacteria responsible for anaplasmosis can infect mammary epithelial cells, resulting in cellular demise and tissue impairment [48].

## Conclusion

This study has been conducted in cattle of District Nowshera and reports a prevalence of *A. marginale* as 13.3% and 12.7% confirmed by microscopy and PCR respectively, with a loss in milk production among infected animals. This regional study is a novel attempted approach focusing on molecular detection, clinical symptoms, and assessment of production loss in a large population of cattle. Four sequences of local msp1b gene are submitted to GenBank, providing the first-ever genetic information about *A. marginale* in this region. The results indicate that there is a need for specific control measures to reduce economic losses to the dairy industry.

## Data Availability

No datasets were generated or analysed during the current study.

## References

[1] Rajput ZI, Hu SH, Arijo AG, Habib M, Khalid M. Comparative study of *Anaplasma* parasites in tick carrying buffaloes and cattle. Journal of Zhejiang University-SCIENCE B. 2005;6(11):1057–62. 16252338 10.1631/jzus.2005.B1057 PMC1390651

[2] Kocan KM, Goff WL, Stiller D, Claypool PL, Edwards W, Ewing SA, Barron SJ. a. Persistence of *A. marginale* (Rickettsiales: *Anaplasma*taceae) in male dermacentor andersoni (Acari: Ixodidae) transferred successively from infected to susceptible calves. J Med Entomol. 1992;29(4):657–68. 1495076 10.1093/jmedent/29.4.657

[3] Kocan KM, Stiller D, Goff WL, Claypool PL, Edwards W, Ewing SA, Barron SJ. b. Development of *A. marginale* in male *Dermacentor andersoni* transferred from parasitemic to susceptible cattle. Am J Vet Res. 1992;53(4):499–507. 1586018

[4] Ge NL, Kocan KM, Blouin EF, Murphy GL. Developmental studies of *A. marginale* (Rickettsiales: *Anaplasmataceae*) in male dermacentor andersoni (Acari: Ixodidae) infected as adults by using nonradioactive in situ hybridization and microscopy. J Med Entomol. 1996;33(6):911–20. 8961639 10.1093/jmedent/33.6.911

[5] Kocan KM, De La Fuente J, Blouin EF, Garcia-Garcia JC. *A. marginale* (Rickettsiales: *Anaplasma*taceae): recent advances in defining host–pathogen adaptations of a tick-borne rickettsia. Parasitology. 2004;129(S1):S285–300. 15938516 10.1017/s0031182003004700

[6] GOA. 2023. Retrieved from: Anaplasmosis: https://www.alberta.ca/anaplasmosis-overview

[7] Sajid MS, Siddique RM, Khan SA, Iqbal Z, Khan MN. Prevalence and risk factors of anaplasmosis in cattle and Buffalo populations of district khanewal, punjab, Pakistan. Global Vet. 2014;12(1):146–53.

[8] Shaukat A, Mehmood K, Shaukat I, Naeem MA, Mehfooz A, Saleem MI, Qureshi AS. Prevalence, haematological alterations and chemotherapy of bovine anaplasmosis in Sahiwal and crossbred cattle of District Faisalabad, Punjab, Pakistan. Pakistan J Zool. 2019;51(6). 10.17582/journal.pjz/2019.51.6.2023.2032.

[9] Atif FA, Khan MS, Iqbal HJ, Arshad GM, Ashraf E, Ullah S. Prevalence of *A. marginale*, *Babesia bigemina* and *Theileria annulata* infections among cattle in Sargodha district, Pakistan. Afr J Agric Res. 2012;7(22):3302–7.

[10] Asif M, Ben Said M, Vinueza RL, Leon R, Ahmad N, Parveen A,… Iqbal F. Seasonal investigation of A. marginale infection in Pakistani cattle reveals hematological and biochemical changes, multiple associated risk factors and msp5 gene conservation. Pathogens 2022;11(11):1261. 10.3390/pathogens11111261 PMC9696815 36365012

[11] Atif FA, Khan MS, Roheen T, Muhammad F, Younus M, Avais M, Ullah S. Seroprevalence of *A. marginale* infection among cattle from three. Pakistan: districts of the northern Punjab; 2013.

[12] Jaimes-Dueñez J, Triana-Chávez O, Mejía-Jaramillo AM. Genetic, host and environmental factors associated with a high prevalence of *A. marginale*. Ticks Tick-borne Dis. 2018;9(5):1286–95. 29793771 10.1016/j.ttbdis.2018.05.009

[13] Isik N, Ekici OD, Sevinc F. The endemic status of *A. marginale* in cattle, in Turkey. Indian J Anim Res. 2018;52(5):750–3.

[14] McHardy N, Simpson RM. Imidocarb dipropionate therapy in Kenyan anaplasmosis and babesiosis. Trop Anim Health Prod. 1974;6:63–70. 4413774 10.1007/BF02380540

[15] Smith RD, Hungerford LL, Armstrong CT. Epidemiologic investigation and control of an epizootic of anaplasmosis in cattle in winter. J Am Vet Med Assoc. 1989;195(4):476–80. 2777688

[16] Potgieter FW. Anaplasmosis. In: Coetzer JAW, Thompson GR, editors. Infectious diseases of Livestock-With special references to Southern Africa. Cape town, South Africa: Oxford University Press; 1994. pp. 408–30.

[17] Aubry P, Geale DW. A review of bovine anaplasmosis. Transbound Emerg Dis. 2011;58(1):1–30. 21040509 10.1111/j.1865-1682.2010.01173.x

[18] Corona B, Dasiel Obregón D, AIfonso Y, Vega E, Díaz A, Martinez S. Tendencies in diagnostic of bovine anaplasmosis. Rev Salud Anim. 2014;36(2):73–9.

[19] Ndung’u LW, Aguirre C, Rurangirwa FR, McElwain TF, McGuire TC, Knowles DP, Palmer GH. Detection of *Anaplasma ovis* infection in goats by major surface protein 5 competitive inhibition enzyme-linked immunosorbent assay. J Clin Microbiol. 1995;33(3):675–9. 7538510 10.1128/jcm.33.3.675-679.1995 PMC228012

[20] Knowles D, Torioni de Echaide S, Palmer G, McGuire T, Stiller D, McElwain T. Antibody against an *A. marginale* MSP5 epitope common to tick and erythrocyte stages identifies persistently infected cattle. J Clin Microbiol. 1996;34(9):2225–30. 8862589 10.1128/jcm.34.9.2225-2230.1996 PMC229221

[21] de la Fuente J, Naranjo V, Ruiz-Fons F, Vicente J, Estrada-Peña A, Almazán C,… Gortázar C. Prevalence of tick-borne pathogens in ixodid ticks (Acari:Ixodidae) collected from European wild boar (Sus scrofa) and Iberian red deer (Cervus elaphus hispanicus) in central Spain. Eur J Wildlife Res. 2004;50(4),187–196.

[22] De La Fuente J, Vicente J, Höfle U, Ruiz-Fons F, De Mera IGF, Van Den Bussche RA,… Gortazar C. Anaplasma infection in free-ranging Iberian red deer in the region of Castilla-La Mancha, Spain. Vet Microbiol 2004;100(3 4):163–173. de Castro JJ. Sustainable tick and tickborne disease control in livestock improvement in developing countries. Vet Parasit 1997;71(2–3):77-97. 10.1016/j.vetmic.2004.02.007 15145495

[23] Visser ES, McGuire TC, Palmer GH, Davis WC, Shkap V, Pipano E, Knowles DP Jr. The *A. marginale* msp5 gene encodes a 19 Kilodalton protein conserved in all recognized *Anaplasma* species. Infect Immun. 1992;60(12):5139–44. 1280624 10.1128/iai.60.12.5139-5144.1992 PMC258289

[24] Munodzana D, McElwain TF, Knowles DP, Palmer GH. Conformational dependence of *A. marginale* major surface protein 5 surface exposed B-cell epitopes. Infect Immun. 1998;66(6):2619–24. 9596725 10.1128/iai.66.6.2619-2624.1998 PMC108247

[25] Hofmann-Lehmann R, Meli ML, Dreher UM, Gönczi E, Deplazes P, Braun U,… Lutz H. Concurrent infections with vector-borne pathogens associated with fatal hemolytic anemia in a cattle herd in Switzerland. J Clin Microbiol 2004;42(8):3775–3780. 10.1128/JCM.42.8.3775-3780.2004 PMC497630 15297529

[26] Lin Q, Rikihisa Y, Felek S, Wang X, Massung RF, Woldehiwet Z. *Anaplasma phagocytophilum* has a functional msp2 gene that is distinct from p44. Infect Immun. 2004;72(7):3883–9. 15213131 10.1128/IAI.72.7.3883-3889.2004 PMC427402

[27] Morzaria SP, Katende J, Musoke A, Nene V, Skilton R, Bishop R. Development of sero-diagnostic and molecular tools for the control of important tick borne pathogens of cattle in Africa. Parassitologia. 1999;41:73–80. 11071549

[28] Ali SI, Qaiser M. To date. Flora of Pakistan. Islamabad: National Herbarium. Pakistan Agricultural Research Council; 1992.

[29] Weather and Climate. Climate and average weather in Nowshera, Khyber Pakhtunkhwa, Pakistan. 2023. Retrieved from https://weatherandclimate.com/pakistan/khyber-pakhtunkhwa/nowshera

[30] Rony SA, Mondal MMH, Begum N, Islam MA, Affroze S. Epidemiology of Hemoprotozoan diseases of cattle in Northern districts of Bangladesh. Bangladesh J Veterinary Med. 2017;15(1):49–56. 10.3329/bjvm.v15i1.34565.

[31] Khattak RM, Rabib AA, Khan Z, Iqbal F, Qayyum M. A report on the high prevalence of *Anaplasma* sp. in buffaloes from two provinces in Pakistan. Ticks Tick-borne Dis. 2013;4(4):267–71. 10.1016/j.ttbdis.2013.01.002. 10.1016/j.ttbdis.2013.04.001 23743023

[32] Kessell A. Bovine haematology and biochemistry. Bovine Med. 2015;146:160.

[33] Ziam H, Kernif T, Saidani K, Kelanemer R, Hammaz Z, Geysen D. Bovine piroplasmosis-anaplasmosis and clinical signs of tropical theileriosis in the plains of Djurdjura (north Algeria). Veterinary Med Sci. 2020;6(4):720–9. 10.1002/vms3.305 PMC7738714 32558239

[34] Bilgic HB, Karagenc T, Simuunza M, Shiels B, Tait A, Eren H, Weir W. Development of a multiplex PCR assay for simultaneous detection of theileria annulata, Babesia Bovis and *A. marginale* in cattle. Exp Parasitol. 2013;133:222–9. 23183165 10.1016/j.exppara.2012.11.005 PMC3650576

[35] Nielsen C. Economic impact of mastitis in dairy cows. Swedish University of Agricultural Sciences. 2009. Retrieved from https://pub.epsilon.slu.se/1968/1/Christel_Nielsen_kappa.pdf

[36] Jongejan F, Uilenberg G. The global importance of ticks. Parasitology. 2004;129(S1):S3-14. 15938502 10.1017/s0031182004005967

[37] Ali J, Khooharo AA, Mirani Z, Siddiqui BN. Farmer’s perception regarding constraints faced in adoption of dairy farming practices in Sindh province, Pakistan. Trop Anim Health Prod. 2019;51:1707–15. 30919321 10.1007/s11250-019-01867-5

[38] Khan A, Saeed K, Nasreen S, Niaz S, Akhtar N. Prevalence of anaplasmosis in cows and buffaloes of district charsadda, Khyber pakhtunkhwa, Pakistan. Glob Vet. 2016;16(5):431–40.

[39] Khan NU, Sarwar MS, Ayaz S, Ali H, Ali A, Khan I,… Rashid G. Prevalence and risk factors analysis associated with anaplasmosis in symptomatic cattle under field conditions in southern Khyber Pakhtoonkhwa, Pakistan. Pure Appl Biol (PAB). 2019;8(4):2119–2127.

[40] Zeb J, Shams S, Din IU, Ayaz S, Khan A, Nasreen N,… Senbill H. Molecular epidemiology and associated risk factors of Anaplasma marginale and Theileria annulata in cattle from North-western Pakistan. Vet Parasitol 2020;279:109044. 10.1016/j.vetpar.2020.109044 32032840

[41] Asif M, Ben Said M, Vinueza RL, Leon R, Ahmad N, Parveen A,… Iqbal F. Seasonal investigation of Anaplasma marginale infection in Pakistani cattle reveals hematological and biochemical changes, multiple associated risk factors and msp5 gene conservation. Pathogens 2022;11(11):1261. 10.3390/pathogens11111261 PMC9696815 36365012

[42] Hamou SA, Rahali T, Sahibi H, Belghyti D, Losson B, Goff W, Rhalem A. Molecular and serological prevalence of *Anaplasma marginale* in cattle of North central Morocco. Res Vet Sci. 2012;93(3):1318–23. 22465306 10.1016/j.rvsc.2012.02.016

[43] Yang J, Han R, Liu Z, Niu Q, Guan G, Liu G, Yin H. Insight into the genetic diversity of Anaplasma marginale in cattle from ten provinces of China. Parasites Vectors. 2017;10:1–7. https://link.springer.com/article/10.1186/s13071-017-2485-x. 29132409 10.1186/s13071-017-2485-x PMC5683237

[44] Radwan ME, Ali AF, El Hamied OA. Epidemiological studies and molecular diagnosis of *A. marginale* in cattle and biochemical changes associated with it in Kaliobia Governorate. American J Res Com. 2013;1(4):247–60.

[45] Badshah F, Ullah K, Kamal M, Rafiq N, Usman T, los Ríos-Escalante D, Said MB. Epidemiological analysis of anaplasmosis in cattle from Khyber Pakhtunkhwa, Pakistan. Vet World 2023;16(11). https://pmc.ncbi.nlm.nih.gov/articles/PMC10750754/. 38152261 10.14202/vetworld.2023.2287-2292 PMC10750754

[46] Vetrivel DA, Serma SPJ, Shilpa JS. A study on predisposing factors for the prevalence of anaplasmosis in dairy cattle. J Entomol Zool Stud. 2017;5(6):1228–32.

[47] Taylor MA, Coop RL, Wall R. Veterinary parasitologyTaylor MA, Coop RL, Wall R. Veterinary parasitology. Wiley; 2015. ISBN 978-1-118-98427-2.

[48] Tabor AE. MSD Manual Veterinary Manual. 2022. Retrieved from https://www.msdvetmanual.com/circulatory-system/blood-parasites/anaplasmosis-in-ruminants

